# Extracellular vesicle-derived AEBP1 mRNA as a novel candidate biomarker for diabetic kidney disease

**DOI:** 10.1186/s12967-021-03000-3

**Published:** 2021-07-31

**Authors:** Yiying Tao, Xing Wei, Yue Yue, Jiaxin Wang, Jianzhong Li, Lei Shen, Guoyuan Lu, Yang He, Shidi Zhao, Fan Zhao, Zhen Weng, Xiahong Shen, Ling Zhou

**Affiliations:** 1 Department of Nephrology, The First Affiliated Hospital of Soochow University, Suzhou, 215006 China; 2 Department of Gastroenterology, The First Affiliated Hospital of Soochow University, Suzhou, 215006 China; 3 MOE Engineering Center of Hematological Disease, Soochow University, Suzhou, 215123 China; 4 Cyrus Tang Hematology Center, Soochow University, Suzhou, 215123 China; 5 National Clinical Research Center for Hematologic Diseases, The First Affiliated Hospital of Soochow University, Suzhou, 215006 China; 6 MOH Key Lab of Thrombosis and Hemostasis, Jiangsu Institute of Hematology, The First Affiliated Hospital of Soochow University, Suzhou, 215006 China; 7 Collaborative Innovation Center of Hematology, Soochow University, Suzhou, 215006 China

**Keywords:** Diabetic kidney disease, Gene expression profiling, Plasma-derived extracellular vesicles, Adipocyte enhancer binding protein 1

## Abstract

**Background:**

A novel and improved methodology is still required for the diagnosis of diabetic kidney disease (DKD). The aim of the present study was to identify novel biomarkers using extracellular vesicle (EV)-derived mRNA based on kidney tissue microarray data.

**Methods:**

Candidate genes were identified by intersecting the differentially expressed genes (DEGs) and eGFR-correlated genes using the GEO datasets GSE30528 and GSE96804, followed by clinical parameter correlation and diagnostic efficacy assessment.

**Results:**

Fifteen intersecting genes, including 8 positively correlated genes, B3GALT2, CDH10, MIR3916, NELL1, OCLM, PRKAR2B, TREM1 and USP46, and 7 negatively correlated genes, AEBP1, CDH6, HSD17B2, LUM, MS4A4A, PTN and RASSF9, were confirmed. The expression level assessment results revealed significantly increased levels of AEBP1 in DKD-derived EVs compared to those in T2DM and control EVs. Correlation analysis revealed that AEBP1 levels were positively correlated with Cr, 24-h urine protein and serum CYC and negatively correlated with eGFR and LDL, and good diagnostic efficacy for DKD was also found using AEBP1 levels to differentiate DKD patients from T2DM patients or controls.

**Conclusions:**

Our results confirmed that the AEBP1 level from plasma EVs could differentiate DKD patients from T2DM patients and control subjects and was a good indication of the function of multiple critical clinical parameters. The AEBP1 level of EVs may serve as a novel and efficacious biomarker for DKD diagnosis.

**Supplementary Information:**

The online version contains supplementary material available at 10.1186/s12967-021-03000-3.

## Background

Diabetic kidney disease (DKD) is one of the most common and severe microvascular complications and is considered one of the most important causes of morbidity and mortality in diabetes patients, accounting for 40% of end-stage kidney disease cases [1, 2]. With a high diabetes prevalence of up to 382 million worldwide, the number of DKD patients is expected to reach a historic high [3]. Therefore, it is necessary to explore the mechanisms involved in the process of DKD development, thereby shedding light on how to better control and prevent this kind of disease. According to the literature [4–6], multiple mechanisms have been reported to be involved in the pathophysiology of DKD, including inflammation, oxidative stress, mesangial hyperplasia and hemodynamics. However, due to the invasive nature of renal biopsy [7] and overestimation of the effectiveness of microalbuminuria quantification [8, 9], a novel and improved methodology is still required for the diagnosis of DKD.

Extracellular vesicles (EVs), including exosomes, microvesicles and other extracellular vesicles, can be secreted by multiple types of cells under normal and disease conditions with a specific average size of approximately 50–200 nm [10, 11], and recent studies have shown that EVs could play important roles in intercellular communication signaling via several of their contents, including mRNAs, noncoding RNAs (miRNAs and long noncoding RNA) and proteins [12, 13]. Studies have shown that urinary EVs and blood-derived EVs could serve as biomarkers for DKD with the features of noninvasiveness and easy collection [14–16]; therefore, which type of EV-derived content is the most effective for diagnosing or monitoring a disease condition is worthy of exploration. Since quantitative reverse transcription polymerase chain reaction (RT-PCR) has been carried out for mRNA level evaluation in most biolabs in current practice, we tried to explore possible EV-derived mRNA biomarkers using plasma samples.

In the present study, we performed bioinformatics analysis to identify eGFR-correlated differentially expressed genes (DEGs) via previously published microarray datasets and validated the efficacy of selected genes in a cohort of DKD patients using plasma-derived EVs to provide a novel biomarker for DKD diagnosis.

## Methods

### Patient inclusion and clinical data collection

A total of 15 DKD patients and 10 healthy control volunteers were recruited from the Department of Nephrology, the First Affiliated Hospital of Soochow University, for plasma sample preparation from May 2019 to August 2020. The National Kidney Foundation Kidney Disease Outcomes Quality Initiative (NKF-KDOQI) guidelines [17] and the Consensus for Prevention and Treatment of Diabetic Kidney Disease 2014 of Chinese Diabetes Society [18] were employed as the diagnostic criteria for DKD patients, and guidelines published in 2010 by the American Diabetes Association (ADA) were used for type 2 diabetes mellitus [19], whereas patients suffering from autoimmune, infectious, hematological, malignant, organic or inflammatory diseases; who underwent renal replacement therapy; who were morbidly obese with body mass index [BMI] ≥ 40 kg/m^2^; or who had cardiovascular diseases accompanying severe complications were excluded. Clinical data were collected from all the patients, and detailed information is shown in Table 1.

**Table 1:** Clinical characteristics of the included subjects

Clinical parameters	Control (n = 10)	T2DM (n = 15)	DKD (n = 15)	P-value
Age (yrs)	47.10 ± 9.27	48.00 ± 10.95	60.13 ± 11.76	0.0048
Gender (female, %)	3 (30.00%)	4(26.67%)	2 (13.33%)	0.5585
BMI	23.75 ± 3.54	26.33 ± 5.77	24.80 ± 2.16	0.3081
SBP (mmHg)	127.10 ± 16.96	123.07 ± 10.07	155.50 ± 20.18	< 0.0001
DBP (mmHg)	82.50 (78.75–85.50)	81.00 (76.00–85.00)	85.00 (81.00–90.00)	0.4273
T2DM duration (yrs)	–	2(0.25–8)	10 (10–20)	< 0.0001
HbA1c (%)	–	10.32 ± 2.24	7.34 ± 1.26	< 0.0001
eGFR (MDRD, ml/min/1.73m^2^)	133.95 ± 32.80	143.73 ± 30.72	18.59 ± 12.64	< 0.0001
eGFR (CKD-EPI, ml/min/1.73m^2^)	107.19 ± 16.53	116.34 ± 13.32	18.05 ± 12.87	< 0.0001
BUN (mmol/L)	4.75 ± 1.10	5.20 ± 1.89	19.17 ± 10.53	< 0.0001
Creatinine (µmol/L)	57.83 ± 11.96	55.70 ± 11.48	465.72 ± 342.07	< 0.0001
TC (mmol/L)	4.71 ± 0.87	4.72 ± 0.83	4.85 ± 1.82	0.9483
TG (mmol)	0.85(0.54–2.34)	1.77(1.57–3.02)	1.49 (1.27–2.95)	0.0791
LDL (mmol/L)	–	2.62 ± 0.84	2.55 ± 1.33	0.8668
HDL (mmol/L)	–	0.93 ± 0.27	0.91 ± 0.17	0.8344
24-h urine protein (g/d)	–	–	6.92 ± 3.10	–
Urine protein to creatinine ratio	–	0.06 ± 0.04	4.87 ± 2.03	< 0.0001
Albumin (g/L)	43.47 ± 1.81	40.42 ± 3.10	30.37 ± 6.26	< 0.0001
Albumin/creatinine ratio (mg/g)	3.44 (0.22–6.49)	–	2692.30 (1251.19–4346.50)	< 0.0001
Urine β2MG (mg/L)	0.21 (0.04–0.27)	–	20.83 (12.07–31.28)	< 0.0001
Urine RBP (mg/L)	0.15 (0.01–0.42)	–	13.40 (7.80–18.54)	< 0.0001
Urine CYC (mg/L)	0.01 (0.01–0.11)	–	1.58 (0.82–2.21)	< 0.0001
Serum CYC (mg/L)	–	0.78 ± 0.07	3.64 ± 1.71	< 0.0001
Hemoglobin (g/L)	137.20 ± 8.64	141.47 ± 13.63	94.40 ± 17.76	< 0.0001
WBC (10^9^/L)	6.15 ± 1.06	6.23 ± 1.04	6.10 ± 1.87	0.9712
Monocytes (10^9^/L)	0.43 ± 0.07	0.45 ± 0.13	0.51 ± 0.28	0.5720
Lymphocytes (10^9^/L)	2.11 ± 0.59	2.12 ± 0.47	1.45 ± 1.20	0.0685
Neutrophils (10^9^/L)	3.45 ± 0.98	3.50 ± 1.03	4.12 ± 1.45	0.2756
hsCRP (mg/L)	–	1.51(1.13–2.88)	1.92(0.61–3.18)	0.9907

Measurement data was expressed as mean ± standard deviation (SD) or median and interquartile range according to normal distribution statue, categorial data was expressed as number and percentages

*BMI* body mass index, *SBP* systolic blood pressure, *DBP* diastolic blood pressure, *T2DM* type II diabetes mellitus, *HbA1c* glycated hemoglobin, *MDRD* The Modification of Diet in Renal Disease, *CKD-EPI* The Chronic Kidney Disease Epidemiology Collaboration, *TC* total cholesterol, *TG* triglycerides, *HDL* high density lipoprotein, *LDL* low-density lipoprotein cholesterol, *BUN* blood urea nitrogen, *RBP* retinol-binding protein, *CYC* cystatin-C, *WBC* White Blood Cell

### Acquisition of microarray data and related clinical data

The mRNA expression and related experimental and clinical data of DKD were downloaded from Gene Expression Omnibus (GEO) (http://www.ncbi.nlm.nih.gov/geo/) using the search terms “diabetic kidney disease”, “diabetic nephropathy” and “expression profiling by array”. The gene expression microarray datasets GSE30528, GSE30529, GSE33744 and GSE96804 were selected and downloaded. The criteria for dataset selection were as follows: human clinical samples with detailed clinical and gene expression information or a mouse model with gene expression information. Among these datasets, GSE96804 was used for differentially expressed gene (DEG) screening, and GSE30528 combined with patient eGFR data downloaded from https://www.nephroseq.org/resource/login.html was used for correlated gene screening. GSE30528, GSE30529 and GSE33744 were used for expression-level validation of the 15 identified eGFR-correlated genes at different tissue composition and experimental mouse model levels. Detailed information on these microarray datasets is listed in Additional file 1: Table S1.

### Candidate gene identification and pathway analysis

GSE96804, with 41 DKD kidney tissue samples and 20 control samples, was used for DEG screening, and the downloaded CEL data were processed with the R package affy [20] for background adjustment, quantile normalization, and median polish summarization, thereby obtaining the gene expression matrix. After performing the gene symbol annotation step using R package hgu133a2.db, the DEGs between DKD and control samples were identified by using R package limma [21] (version 3.40.6) and criteria of fold change > 1.5 and adjusted p-values < 0.05. GSE30528, with gene expression data from DKD-derived kidney tissue and eGFR data, was used for eGFR-correlated gene screening with the Pearson correlation method. Moreover, a Venn diagram was used to determine the intersecting genes from the above DEGs and eGFR-correlated genes, followed by related expression level pattern validation using the data from GSE30528, GSE30529 and GSE33744. The flowchart of the data processing procedures is shown in Additional file 2: Fig. S1. In addition, the R package pheatmap [22] (version 1.0.12) was employed for heatmap and volcano map preparation for DEGs from GSE96804, whereas the R package clusterProfiler [23] (version 3.14.3) was used for Gene Ontology and KEGG pathway analysis for DEGs and eGFR-correlated genes. The present study was approved by the Ethics Committee of the First Affiliated Hospital of Soochow University and was carried out in accordance with the Declaration of Helsinki. Written informed consent was provided by all the included subjects.

### EVs isolation and characterization

Five milliliters of anticoagulant-treated venous blood was collected from the abovementioned DKD and control subjects for plasma isolation, and exosomes were isolated from whole blood with sequential centrifugation and an ExoQuick™ Plasma Prep and Exosome Precipitation Kit (Cat# EXOQ5TM-1, SBI System Biosciences, Mountain View, CA, USA) according to the manufacturer’s instructions. Briefly, sequential centrifugation at 1200 g for 10 min, 3000 g for 20 min, and 10,000 g for 30 min was performed on the whole blood to remove the blood cells, dead cells and large vesicle to obtain the plasma supernatant, followed by mixing with ExoQuick Exosome Precipitation Solution at the ratio of 4:1 and sequential centrifugation at 1500 g for 30 min and 1500 for 5 min; then, the precipitated pellet containing exosomes was obtained and resuspended in 1 × PBS for further study. EV characterization was carried out using electron microscopy for morphology observation, dynamic light scattering (DLS) for particle size distribution and western blotting for protein marker identification. For electron microscopy, exosomes resuspended in 2 µg/ml PBS were fixed with 2% paraformaldehyde (PFA) at a ratio of 1:1 and loaded onto transmission electron microscopy grids until dry, followed by 1% glutaraldehyde fixation, purified water washing and 1% phosphotungstic acid (PTA) staining. The samples were observed using a Tecnai G2 Spirit BioTwin transmission electron microscope (FEI company, Hillsboro, OR, USA). For DLS, exosomes resuspended in 2 µg/ml PBS were diluted with PBS at a ratio of 1:1000 and then subjected to NanoSight LM10 (Malvern Panalytical, UK) for analysis. For western blotting, exosomes and exosome-depleted supernatant (EDS; negative control) from DKD patients and control subjects were processed for analysis. Forty-five micrograms of exosomes and the same volume of EDS were loaded for SDS-PAGE, followed by PVDF membrane transfer, nonfat milk blocking, primary and secondary antibody incubation, and chemiluminescence exposure. The primary antibodies for Flotillin-1 and GM130 were obtained from Cell Signaling Technology (Cat. no: 74220), and anti-CD63 was obtained from Abcam (Cat. no: ab252919).

### EV-derived RNA extraction and evaluation of gene expression levels

RNA extraction from EVs was performed using TRIzol™ Reagent (Thermo Fisher, CA, USA) according to the manufacturer’s instructions. After RNA quantification and qualification by a NanoDrop 2000 (Thermo Fisher, CA, USA), reverse transcription and quantitative PCR were carried out using a RevertAid RT Reverse Transcription Kit (Thermo Fisher, CA, USA) and SYBR Green qPCR Mix (Beyotime, Nantong, China), respectively, to evaluate the expression level of AEBP1. The expression of GAPDH was selected as the internal control according to previous publications [24, 25]. The primer sequences were as follows: AEBP1: Forward: 5’-ACCCACACTGGACTACAATGA-3’, Reverse: 5’-GTTGGGGATCACGTAACCATC-3’; GAPDH: Forward: 5’-GCAAATTCCATGGCACCGT-3’, Reverse: 5’-TCGCCCCACTTGATTTTGG-3’.

### Statistical analysis

Statistical analysis was carried out using R version 3.6.0. Measurement data are expressed as the mean ± standard deviation (SD), and categorical data are expressed as numbers and percentages. One-way ANOVA and Kruskal–Wallis H tests for parametric and nonparametric continuous variables were used to compare three groups, while unpaired t tests and Mann–Whitney U tests for parametric and nonparametric continuous variables were used for 2-group comparisons. Correlation analysis between the clinical parameters and AEBP1 expression level was performed using Pearson correlation. The diagnostic efficacy was calculated by receiver operator characteristic (ROC) curves and area under the curve (AUC) (survivalROC and ROCR R packages). Unless specifically mentioned, P < 0.05 was considered statistically significant.

## Results

### Identification of candidate genes and pathway analysis

**Fig. 1:**
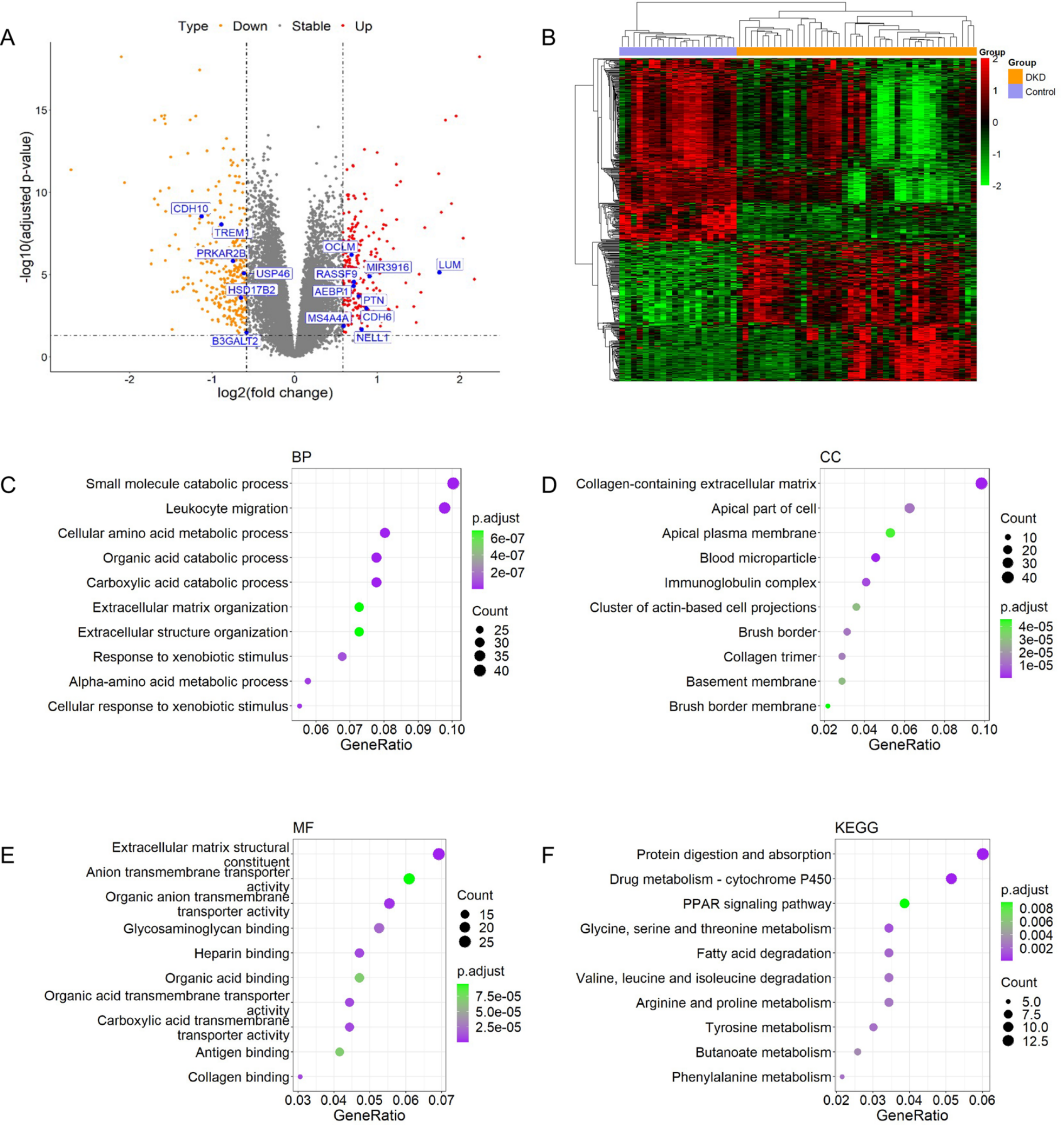
Differentially expressed genes (DEGs) screening and pathway analysis using microarray data from GSE96804. **A** Volcano plot showed up- and down-regulated DEGs. **B** Heatmap indicated up-(red) and down(green)-regulated DEGs stratified by control subjects (orange) and diabetic kidney disease (DKD) patients (blue). **C**–**E** GO pathway analysis using DEGs and figures about biological process (BP), cellular components (CC) and molecular function (MF) were respectively exhibited. **F** KEGG pathway analysis using DEGs

**Fig. 2:**
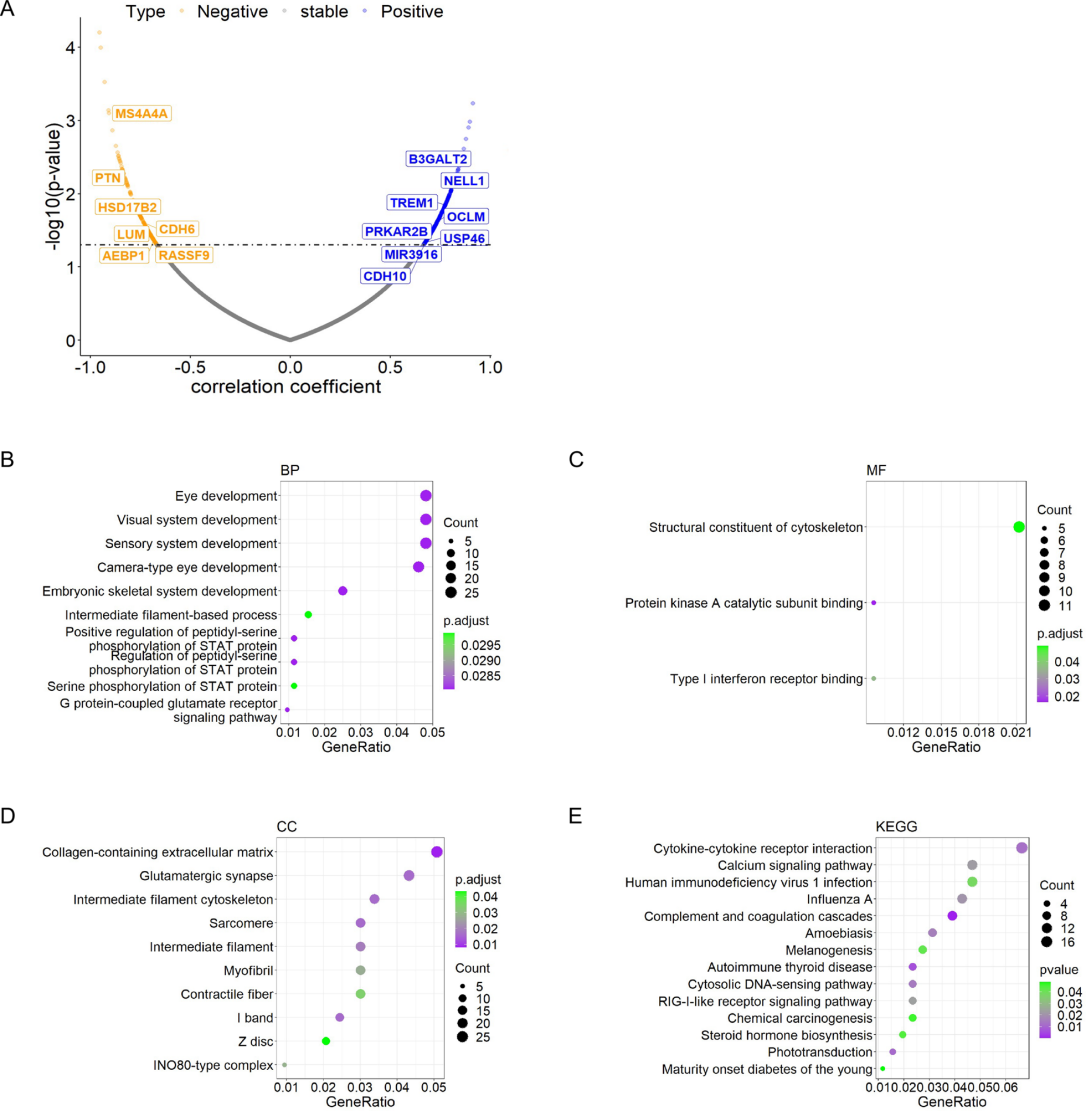
eGFR correlated genes screening and pathway analysis using microarray data from GSE30528. **A** Pearson correlation was performed to obtain the eGFR correlated genes. **B**–**D** GO pathway analysis using eGFR correlated genes and figures about biological process (BP), cellular components (CC) and molecular function (MF) were respectively exhibited. **E** KEGG pathway analysis using eGFR correlated genes

**Fig. 3:**
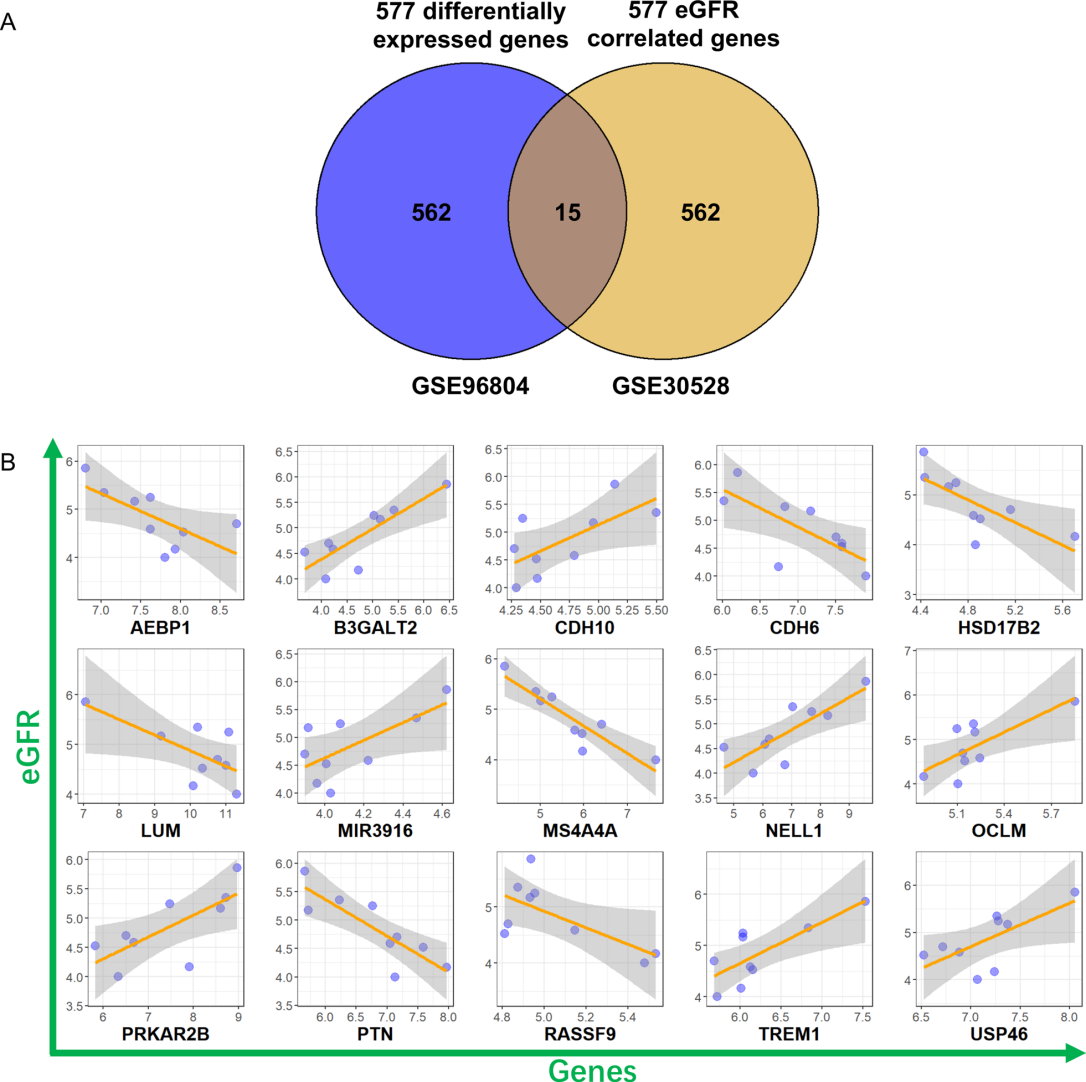
Venn diagram of the intersected genes and their correlation analysis with eGFR data. **A** A total of 15 intersected genes were found via taking intersection of DEGs from GSE96804 and eGFR correlated genes from GSE30528. **B** Correlation analysis between these 15 intersected genes and eGFR was performed, and 8 positive (B3GALT2, CDH10, MIR3916, NELL1, OCLM, PRKAR2B, TREM1, USP46) and 7 negative (AEBP1, CDH6, HSD17B2, LUM, MS4A4A, PTN, RASSF9) correlated genes was identified

To identify candidate genes for further analysis in EVs, we first obtained DEGs from the GSE96804 dataset and constructed volcano plot (Fig. 1A), heatmap (Fig. 1B), GO pathway (Fig. 1C-E) and KEGG pathway (Fig. 1F) figures. A total of 577 DEGs were identified, including 251 upregulated genes and 326 downregulated genes. GO pathway analysis revealed small-molecule catabolic processes, leukocyte migration, cellular amino acid metabolic process as the top 3 biological processes (BPs) (Fig. 1C); collagen-containing extracellular matrix, apical part of cell and apical plasma membrane as the top 3 cellular components (CCs) (Fig. 1D); and extracellular matrix structural constituent, anion transmembrane transporter activity and organic anion transmembrane transporter activity as the top 3 molecular functions (MFs) (Fig. 1E). Protein digestion and absorption, drug metabolism-cytochrome p450, and the PPAR signaling pathway were the top 3 KEGG pathways (Fig. 1F). eGFR-correlated genes (577 genes, including 370 positively correlated and 207 negatively correlated) were also calculated using the GSE30528 dataset and patient eGFR data, and the results are shown in Fig. 2A. Moreover, GO pathway analysis of the top 10 BPs, CCs and MFs (Fig. 2B-D) and the top 10 KEGG pathways (Fig. 2E) was also performed. Then, the 15 intersecting genes from eGFR-correlated genes and DEGs were confirmed (Fig. 3A), including 8 positively correlated genes, B3GALT2, CDH10, MIR3916, NELL1, OCLM, PRKAR2B, TREM1 and USP46, and 7 negatively correlated genes, AEBP1, CDH6, HSD17B2, LUM, MS4A4A, PTN and RASSF9. Moreover, the correlations between these 15 genes and eGFR were also explored (Fig. 3B, Table 2 and Additional file 1: Table S2).

**Table 2:** Correlation coefficient and gene function of 15 intersected genes

Gene names	R value	P value
AEBP1	− 0.68	0.042
B3GALT2	0.83	0.0047
CDH10	0.67	0.046
CDH6	− 0.73	0.025
HSD17B2	− 0.74	0.021
LUM	− 0.69	0.041
MIR3916	0.67	0.047
MS4A4A	− 0.91	0.00079
NELL1	0.80	0.0092
OCLM	0.73	0.023
PRKAR2B	0.72	0.028
PTN	− 0.84	0.0043
RASSF9	− 0.67	0.049
TREM1	0.77	0.014
USP46	0.68	0.046

### Identification of the expression pattern of 15 intersecting genes

**Fig. 4:**
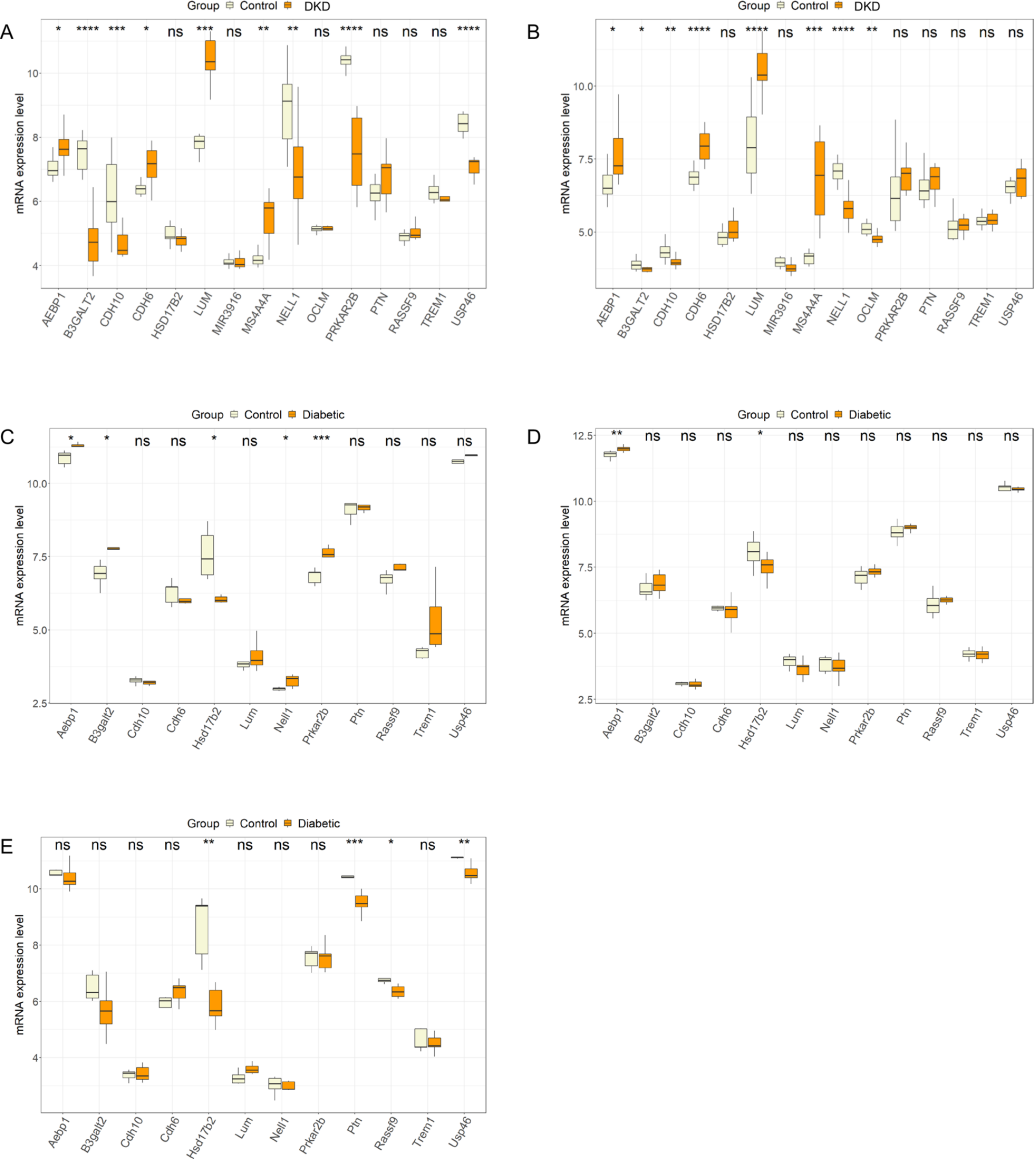
Expression pattern of the 15 intersected genes in different microarray datasets. **A** DKD and control glomeruli samples from GSE30528, **B** DKD and control tubuli samples from GSE30529, **C** Glomeruli samples from Diabetic BKS db/db mice from GSE33744, **D** Glomeruli samples from Diabetic DBA-STZ mice from GSE33744. **E** Glomeruli samples from Diabetic BKS db/db eNOS −/− mice from GSE33744. ns: not significant, *p < 0.05, **p < 0.01, ***p < 0.001

We further evaluated the expression pattern of 15 intersecting genes in 3 microarray datasets, GSE30528, GSE30529 and GSE33744. The up- and downregulated trends of the 15 intersecting genes in human DKD and control glomeruli samples from GSE30528, human DKD and control tubule samples from GSE30529, glomeruli samples from diabetic BKS db/db mice from GSE33744, glomeruli samples from diabetic DBA-STZ mice from GSE33744 and glomeruli samples from diabetic BKS db/db eNOS −/− mice from GSE33744 are shown in Fig. 4A–E and Table 3. Because of the consistent expression pattern of AEBP1 in 4 of the above human and mouse kidney-derived tissues, it was selected for further analysis. Moreover, considering filtration between blood and glomeruli in renal physiology, it is reasonable to use plasma-derived samples.

**Table 3:** Significantly up and down-regulated genes in different microarray datasets among the 15 intersected genes

Microarray set no.	Sample type	Up	Down
GSE30528	Human DKD and control glomeruli samples	AEBP1, CDH6, LUM, MS4A4A	B3GALT2, CDH10, NELL1, PRKAR2B, USP46
GSE30529	Human DKD and control tubuli samples	AEBP1, CDH6, LUM, MS4A4A, PRKAR2B	B3GALT2, CDH10, NELL1, OCLM
GSE33744	Glomeruli samples from Diabetic BKS db/db mice	AEBP1, B3GALT2, NELL1, PRKAR2B	HSD17B2
GSE33744	Glomeruli samples from Diabetic DBA-STZ mice	AEBP1	HSD17B2
GSE33744	Glomeruli samples from Diabetic BKS db/db eNOS −/− mice		HSD17B2, PTN, RASSF9, USP46

### Characterization of EVs from DKD and T2DM patients and controls and evaluation of the expression level of AEBP1

**Fig. 5:**
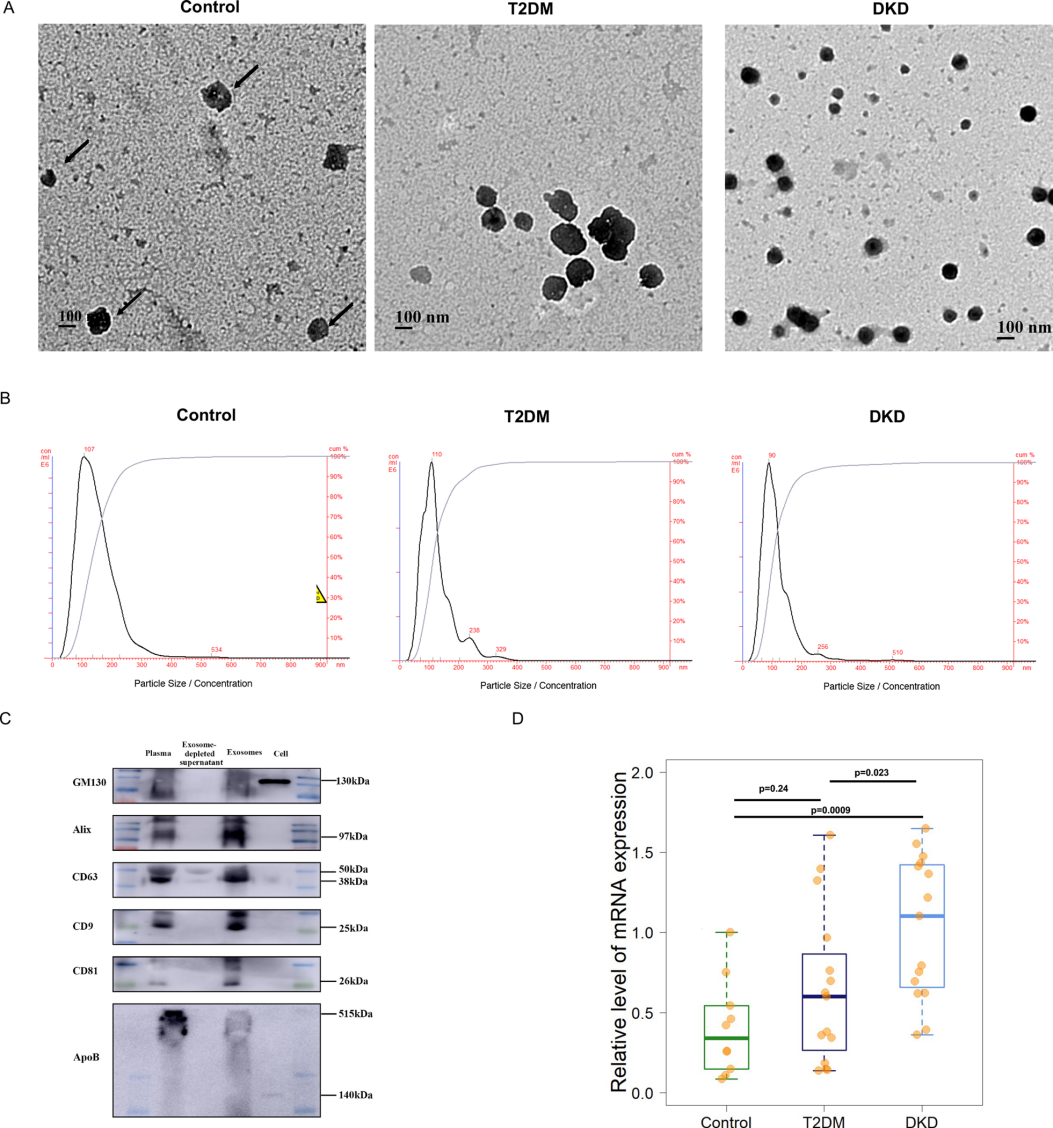
Characterization of extracellular vesicle (EV) from DKD patients, T2DM patients and control subjects and evaluation of the expression level of AEBP1. **A** Representative electronic microscope photograph of EVs from control (with arrow head) subject, T2DM patient (black stained) and DKD patient (black stained). **B** Dynamic light scattering (DLS) results for exosome particles distribution from control subject, T2DM patient and DKD patient. **C** Western-blotting results for exosome marker evaluation in control, T2DM and DKD derived exosomes, whereas PBS as negative control. **D** Significantly increased level of AEBP1 mRNA could be found in DKD patient derived exosome compared to T2DM patient and control subjects

According to the electron microscopy (Fig. 5A), DLS (Fig. 5B) and western blotting results (Fig. 5C), we obtained round morphology exosomes with a mean size of 107 nm in control subjects, 128 nm in T2DM patients and 90 nm in DKD patients with positive expression of CD9, CD63 and CD81 and negative expression of GM130 and ApoB, which is consistent with previous reports [10, 11]. We further evaluated the expression levels of AEBP1 using exosome RNA from DKD and control subjects, and significant AEBP1 expression was found in DKD patients compared to T2DM patients (p = 0.023, Fig. 5D) or control subjects (p = 0.0009, Fig. 5D). These results suggested the possible involvement of AEBP1 in the disease process of DKD.

### Correlation between the expression level of EV-derived AEBP1 and clinical indexes of DKD and exploration of the diagnostic efficacy of AEBP1

**Fig. 6:**
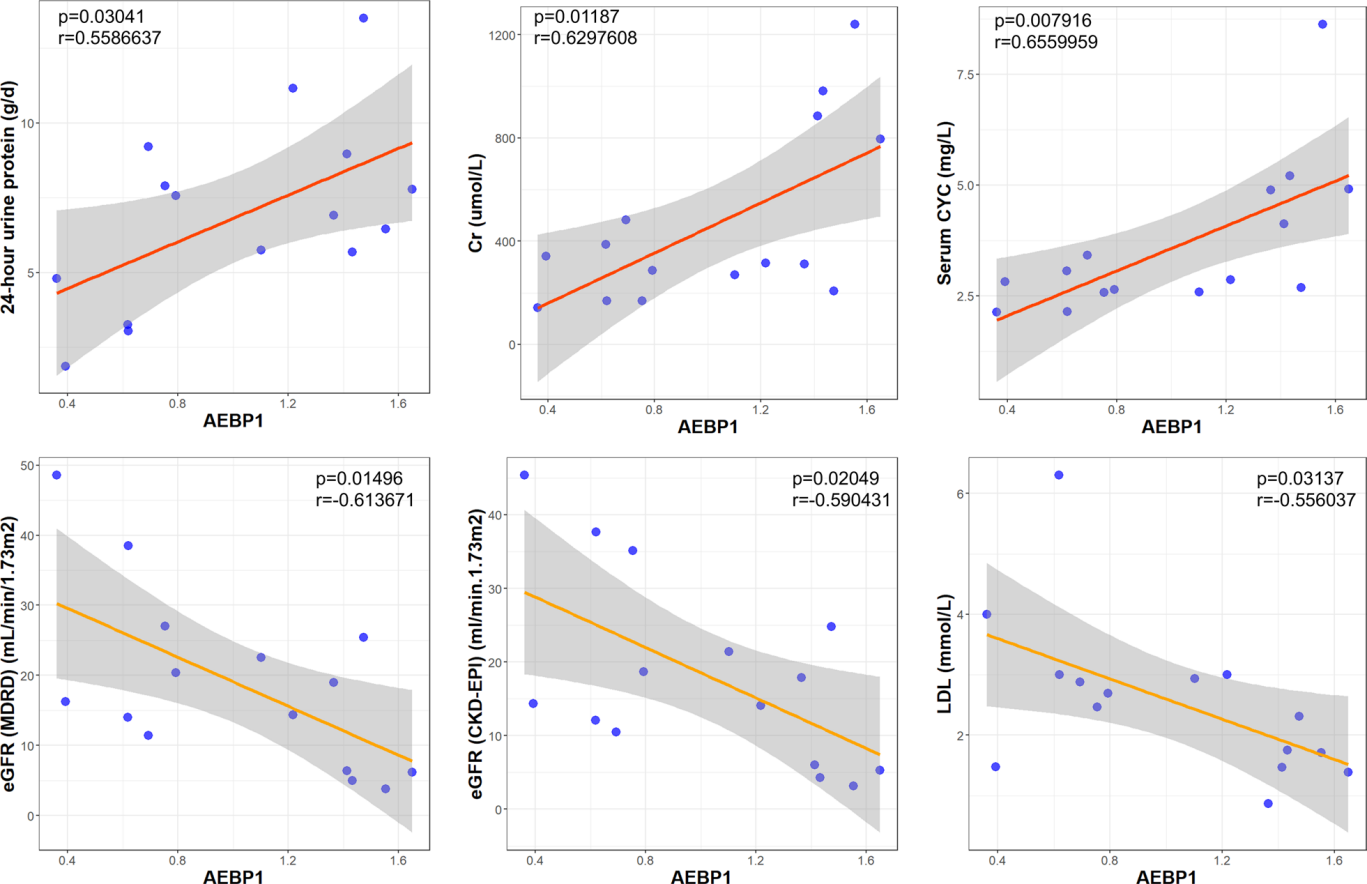
Correlation analysis between the expression level of extracellular vesicle (EV) derived AEBP1 and the clinical parameters in DKD patients. Significant correlation could be found the expression level of AEBP1 and 24-h urine protein, Creatinine (Cr), serum cystatin-C (CYC), eGFR (MDRD), eGFR (CKD-EPI) and LDL

**Fig. 7:**
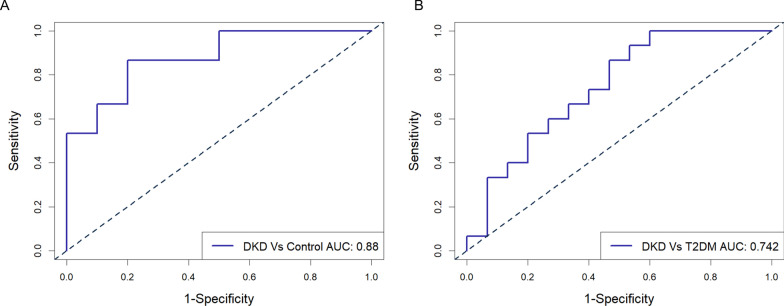
Receiver operator characteristic (ROC) curve evaluation of the diagnostic accuracy of AEBP1 in differentiation of DKD patients, T2DM patients and healthy controls. **A** DKD and healthy controls, Area under curve (AUC) = 0.880, p < 0.001. **B** DKD and T2DM, Area under curve (AUC) = 0.742, p = 0.009

Since we observed the possible involvement of AEBP1 in the disease process of DKD, we evaluated the correlation between the expression level of EV-derived AEBP1 and clinical parameters of DKD. The results showed that the expression level of EV-derived AEBP1 was positively correlated with Cr, 24-h urine protein and serum CYC and negatively correlated with eGFR and LDL (Fig. 6) but not age, BMI, SBP, DBP, HbA1c (%), BUN, TC, TGs, HDL, plasma creatinine, albumin, albumin/creatinine ratio, urine β2MG, urine RBP, urine CYC, hemoglobin, WBC, monocytes, lymphocytes or neutrophils (Table 4 and Additional file 3: Figure S2). Moreover, a trend of correlation could be found between AEBP1 level and hsCRP (r = 0.474, p = 0.074) (Table 4 and Additional file 3: Figure S2). Furthermore, we had to point out that although a significant difference in age and SBP could be found between control and DKD patients, no correlation of these 2 indexes with the AEBP1 level could be found (Additional file 4: Figure S3). In addition, good diagnostic efficacy for DKD was also found using the expression level of exosome-derived AEBP1, with an AUC of 0.880 and p < 0.001 for differentiating healthy controls and an AUC of 0.742 and p = 0.009 for differentiating T2DM according to the ROC curve (Fig. 7). The optimal cutoff value for AEBP1 mRNA level was 0.511 (specificity: 0.800, sensitivity: 0.867) and 0.403 (specificity: 0.533, sensitivity: 0.867) for control vs. DKD and T2DM vs. DKD, respectively.

**Table 4:** Correlation between exosome AEBP1 level and clinical parameters in DKD patients

Clinical parameters	Exosome AEBP1 level	
	R value	P value
Age (yrs)	− 0.283	0.307
BMI	− 0.242	0.384
SBP (mmHg)	− 0.309	0.263
DBP (mmHg)	− 0.140	0.619
HbA1c (%)	− 0.062	0.827
eGFR (MDRD, ml/min/1.73m^2^)	− 0.614	**0.0015**
eGFR (CKD-EPI, ml/min/1.73m^2^)	− 0.59	**0.020**
BUN (mmol/L)	0.298	0.280
Creatinine (µmol/L)	0.629	**0.012**
TC (mmol/L)	− 0.170	0.545
TG (mmol)	− 0.161	0.567
LDL (mmol/L)	− 0.556	**0.031**
HDL (mmol/L)	− 0.034	0.905
24-h urine protein (g/d)	0.559	**0.0304**
Urine protein to creatinine ratio	− 0.079	0.778
Albumin (g/L)	− 0.335	0.222
Albumin/creatinine ratio (mg/g)	0.258	0.374
Urine β2MG (mg/L)	− 0.003	0.993
Urine RBP (mg/L)	0.357	0.255
Urine CYC (mg/L)	− 0.189	0.667
Serum CYC (mg/L)	0.656	**0.008**
Hemoglobin (g/L)	− 0.424	0.116
WBC (10^9^/L)	− 0.101	0.719
Monocytes (10^9^/L)	− 0.068	0.810
Lymphocytes (10^9^/L)	− 0.157	0.576
Neutrophils (10^9^/L)	− 0.041	0.885
hsCRP (mg/L)	0.474	0.074

Bold indicates *p* < 0.05

*BMI* body mass index, *SBP* systolic blood pressure, *DBP* diastolic blood pressure, *T2DM* type II diabetes mellitus, *HbA1c* glycated hemoglobin, *MDRD* The Modification of Diet in Renal Disease, *CKD-EPI* The Chronic Kidney Disease Epidemiology Collaboration, *TC* total cholesterol, *TG* triglycerides, *HDL* high density lipoprotein, *LDL* low-density lipoprotein cholesterol, *BUN* blood urea nitrogen, *RBP* retinol-binding protein, *CYC* cystatin-C, *WBC* White Blood Cell

## Discussion

EVs, secreted by almost all cell types, are nanoscale lipid bilayer spherical shapes containing various receptors, proteins, genetic materials (DNA, mRNA and miRNAs, etc.) with intercellular communication abilities that are made possible by three major mechanisms: receptor-ligand interactions, direct plasma membrane fusion and endocytosis [26, 27]. With the deep recognition of EVs, they have been discovered in multiple biological fluids, including blood, urine, saliva and cerebrospinal fluid, and because these sample types are common in clinical practice, the employment of EVs in disease diagnosis and assessment is reasonable [16, 28]. Eissa et al. reported the employment of urinary EV-derived miR-15b, miR-34a, miR-636, miR-133b, miR-342 and miR-30a as biomarkers in type 2 DN patients [29, 30]. Zubiri et al. suggested the use of urinary bikunin precursor and histone-lysine N-methylthransferase (KHMT) as biomarkers for DKD patients [31], whereas Rossi et al. [32] found that urinary EV-derived AQP2 and AQP5 could be used as biomarkers for DKD patients. Since the use of a single miRNA could only exhibit limited accuracy and specificity as a disease biomarker and the detection of enzyme activity, such as KHMT, is relatively expensive, the possible detection of specific EV-derived mRNA molecules could be a better choice. Furthermore, the vascular property of glomeruli and the adequate number of EVs derived in blood make it reasonable to explore possible novel biomarkers in plasma-derived exosomes.

In the present study, based on the shared microarray datasets in GEO and the available patient eGFR from Nephroseq v5, we first identified eGFR-correlated DEGs from human glomeruli samples, verified these genes in multiple human and mouse models, and then identified AEBP1 as a novel and effective candidate gene for further experiments. Due to the easy collection of blood samples without renal biopsy and the maturity of the methodology of EV isolation, we checked the expression of AEBP1 using EV-derived RNA, and the results showed a significant increase in AEBP1 expression in DKD patients compared to that in control subjects. Moreover, we found that AEBP1 levels were negatively correlated with eGFR and LDL and positively correlated with Cr, 24-h urine protein, and serum CYC. In addition, good diagnostic efficacy could be achieved using EV-derived AEBP1 levels with an AUC of 0.880. This is the first report of EV-derived AEBP1 levels as a novel biomarker for DKD diagnosis.

The advantages of plasma vesicle mRNA markers are as follows: Since the changes in renal pathology occur earlier than reflected by the existing indexes, such as eGFR and urinary albumin, the plasma vesicle mRNA could be an early marker of pathology. Urinary samples could be affected by multiple factors, including drinking water, food consumption and urinary tract infection. Plasma-derived EVs are stable and adequate and could reflect versatile cell type derivates.

Adipocyte enhancer binding protein 1 (AEBP1) was first found in adipocytes and has been reported to be involved in multiple biological processes, including cell differentiation [33], adipogenesis [34], cholesterol homeostasis and inflammation [35]. In the correlation analysis, we found a correlation between AEBP1 and LDL levels and a trend of correlation between AEBP1 levels and hsCRP in DKD patients, which is consistent with the above-stated role of AEBP1 in cholesterol homeostasis and inflammation [35]. Moreover, AEBP1 was reported to be expressed in macrophages with inflammatory-enhancing effects [35, 36]. However, we did not find a correlation between AEBP1 levels and WBCs, monocytes, lymphocytes or neutrophils, which further strengthens the role of AEBP1 in inflammatory cell differentiation but not in cell number increase. Furthermore, AEBP1 is also considered to be involved in multiple disease and pathological processes, and increased levels of AEBP1 accompanying several signal pathway dysfunctions (such as NF-κB [37], Hedgehog [38], etc.) could be found in liver fibrosis [39], Alzheimer’s disease [40], breast epithelial cell hyperproliferation [38], abdominal aortic aneurysm [37] and colorectal cancers [41]. In addition, AEBP1 mutation could also result in hereditary connective tissue diseases [42]. Here, we detected AEBP1 mRNA in plasma-derived exosomal RNA and confirmed its upregulated pattern in DKD patients. According to previous studies, upregulated AEBP1 could be regulated by the transcription factor CREB and the PI3K/Akt pathway in melanoma cells [43], and activation of the PI3K/Akt signaling cascade could be found in diabetic patient-derived peripheral blood mononuclear cells (PBMCs) [44]. Therefore, these PBMCs could be the possible origin of the AEBP1-containing exosomes. In addition, considering the inflammatory state of the kidney and the expression of AEBP1 in glomerular tissues, upregulation of AEBP1 levels in plasma could also result from kidney-derived cells, which is consistent with the microarray results showing upregulation of AEBP1 levels in glomerular tissues.

In addition to AEBP1, we also found 14 other eGFR-correlated genes here, most of which lack functional studies with only microarray information. According to previous studies, B3GALT2 was previously shown to be downregulated in a mouse model of diabetic nephropathy [45], and CDH6 was upregulated in renal fibrosis mice [46]. Upregulated HSD17B2 and LUM were found in diabetic db/db mice [47] and human-derived kidney tissues, respectively [48]. NELL1 is a target antigen in malignancy-associated membranous nephropathy [49]. PTN triggers inflammation and increases peritoneal permeability, leading to peritoneal fibrosis [50]. TREM-1 plays an important role in high-glucose-induced macrophage phenotypic transformation during the progression of DKD [51], whereas no report has been found on the CDH10, MIR3916, MS4A4A, OCLM, RASSF9 or USP46 genes in DKD.

There are several limitations in present study. First, the relatively small number of DKD patients could result in bias in the results on the diagnostic efficacy and difference comparison between the patients and normal subjects. Second, the difficulties that were faced in the recruitment of diabetes patients in our department could result in an inability to determine the AEBP1 level in these patients. Third, due to the lack of basic cell or animal models, the detailed mechanisms by which exosomes with high AEBP1 expression are involved in the disease process of DKD have not been fully explored. Therefore, further study with a larger number and more complete types of patients could be helpful for testing the results obtained here. In addition, a mechanistic study of AEBP1 might also be performed to elucidate its possible role in DKD.

## Conclusions

In conclusion, our results demonstrated that plasma EV-derived AEBP1 levels are capable of differentiating DKD patients from healthy controls and also exhibited good functions for indicating multiple critical clinical parameters. The plasma EV-derived AEBP1 level may serve as a novel and effective biomarker for DKD diagnosis.

## Supplementary Information

**Additional file 1: Table S1.** Detailed information of the included microarray datasets. **Table S2.** Correlation coefficient and gene function of 15 intersected genes.

**Additional file 2: Figure S1.**

**Additional file 3: Figure S2.**

**Additional file 4: Figure S3.**

## Data Availability

All data generated or analysed during this study are included in this published article [and its additional files].
