# Supplementary material for: Extracellular vesicle-derived AEBP1 mRNA as a novel candidate biomarker for diabetic kidney disease
Source: J Transl Med. 2021 Jul 31;19:326. doi: 10.1186/s12967-021-03000-3 (PMC8325821; doi:10.1186/s12967-021-03000-3)
Supplement: Supplementary file 1 — Additional file 1: Table S1. Detailed information of the included microarray datasets. Table S2. Correlation coefficient and gene function of 15 intersected genes. [file 12967_2021_3000_MOESM1_ESM.docx]

**Table S1.** Detailed information of the included microarray datasets

| Series | Platform | Platform Name | Sample information |
| --- | --- | --- | --- |
| GSE96804 | [GPL17586](https://www.ncbi.nlm.nih.gov/geo/query/acc.cgi?acc=GPL17586) | Affymetrix® microarray platform (Human Transcriptome Array 2.0, HTA 2.0) | 41 glomeruli from kidneys with diabetic nephropathy (DN) and 20 glomeruli from the unaffected portion of tumor nephrectomies |
| GSE30528 | [GPL571](https://www.ncbi.nlm.nih.gov/geo/query/acc.cgi?acc=GPL571) | [HG-U133A_2] Affymetrix Human Genome U133A 2.0 Array | 9 DKD and 13 control glomeruli samples |
| GSE30529 | [GPL571](https://www.ncbi.nlm.nih.gov/geo/query/acc.cgi?acc=GPL571) | [HG-U133A_2] Affymetrix Human Genome U133A 2.0 Array | 10 DKD and 12 control tubuli samples |
| GSE33744 | [GPL1261](https://www.ncbi.nlm.nih.gov/geo/query/acc.cgi?acc=GPL1261) | [Mouse430_2] Affymetrix Mouse Genome 430 2.0 Array | glomeruli samples from 22 patients with early type 2 DN and 18 healthy controls, and glomerular RNA from three mouse models including streptozotocin DBA/2 mice, db/db C57BLKS, and eNOS-deficient C57BLKS db/db mice |

**Table S2.** Correlation coefficient and gene function of 15 intersected genes

| Gene names | R value | P value | Gene function |
| --- | --- | --- | --- |
| AEBP1 | -0.68 | 0.042 | A transcriptional repressor and play a role in adipogenesis and smooth muscle cell differentiation, and functions in wound healing and abdominal wall development. Its overexpression is associated with glioblastoma. |
| B3GALT2 | 0.83 | 0.0047 | This gene functions in N-linked glycoprotein glycosylation and shows strict donor substrate specificity for UDP- galactose. |
| CDH10 | 0.67 | 0.046 | This gene mediates calcium-dependent cell-cell adhesion and is predominantly expressed in brain and is putatively involved in synaptic adhesions, axon outgrowth and guidance. Mutations in this gene may be associated with lung squamous cell carcinoma and colorectal cancer in human patients. |
| CDH6 | -0.73 | 0.025 | This gene is a calcium-dependent cell adhesion protein and interacts with itself in a homophilic manner in connecting cells, thereby contributing to the sorting of heterogeneous cell types. |
| HSD17B2 | -0.74 | 0.021 | catalyzing the interconversion of testosterone and androstenedione, as well as estradiol and estrone. |
| LUM | -0.69 | 0.041 | This gene is the major keratan sulfate proteoglycan of the cornea but is also distributed in interstitial collagenous matrices throughout the body and may regulate collagen fibril organization and circumferential growth, corneal transparency, and epithelial cell migration and tissue repair. |
| MIR3916 | 0.67 | 0.047 | MIR3916 is an RNA Gene, and is affiliated with the miRNA class. |
| MS4A4A | -0.91 | 0.00079 | This gene is expressed in hematopoietic cells and nonlymphoid tissues and associated diseases include [Syndromic X-Linked Intellectual Disability Cabezas Type](http://www.malacards.org/card/syndromic_x_linked_intellectual_disability_cabezas_type) and [Froelich Syndrome](http://www.malacards.org/card/froelich_syndrome). |
| NELL1 | 0.80 | 0.0092 | This gene is involved in cell growth regulation and differentiation and associated diseases include [Craniosynostosis](http://www.malacards.org/card/craniosynostosis) and [Synostosis](http://www.malacards.org/card/synostosis). |
| OCLM | 0.73 | 0.023 | NF |
| PRKAR2B | 0.72 | 0.028 | This gene plays an important role in regulating energy balance and adiposity, may mediate the gene induction and cataleptic behavior induced by haloperidol, and associated diseases include [Carney Complex Variant](http://www.malacards.org/card/carney_complex_variant) and [Spinocerebellar Ataxia, Autosomal Recessive 16](http://www.malacards.org/card/spinocerebellar_ataxia_autosomal_recessive_16). |
| PTN | -0.84 | 0.0043 | This gene plays significant roles in cell growth and survival, cell migration, angiogenesis and tumorigenesis, and associated diseases include [Peyronie's Disease](http://www.malacards.org/card/peyronies_disease) and [Nasal Cavity Olfactory Neuroblastoma](http://www.malacards.org/card/nasal_cavity_olfactory_neuroblastoma). |
| RASSF9 | -0.67 | 0.049 | This gene associates with peptidylglycine alpha-amidating monooxygenase, and may be involved with the trafficking of this enzyme through secretory or endosomal pathways,and associated diseases include [Isolated Growth Hormone Deficiency, Type Ii](http://www.malacards.org/card/isolated_growth_hormone_deficiency_type_ii). |
| TREM1 | 0.77 | 0.014 | This gene amplifies neutrophil and monocyte-mediated inflammatory responses triggered by bacterial and fungal infections by stimulating release of pro-inflammatory chemokines and cytokines, as well as increased surface expression of cell activation markers,and associated diseases include [Suppurative Cholangitis](http://www.malacards.org/card/suppurative_cholangitis) and [Acute Cholangitis](http://www.malacards.org/card/acute_cholangitis). |
| USP46 | 0.68 | 0.046 | This gene functions as deubiquitinating enzymes,and associated diseases include [Tracheal Cancer](http://www.malacards.org/card/tracheal_cancer). |

NF: not found in genecards.org
