# Supplementary figures and images for: Extracellular vesicle-derived AEBP1 mRNA as a novel candidate biomarker for diabetic kidney disease
Source: J Transl Med. 2021 Jul 31;19:326. doi: 10.1186/s12967-021-03000-3 (PMC8325821; doi:10.1186/s12967-021-03000-3)

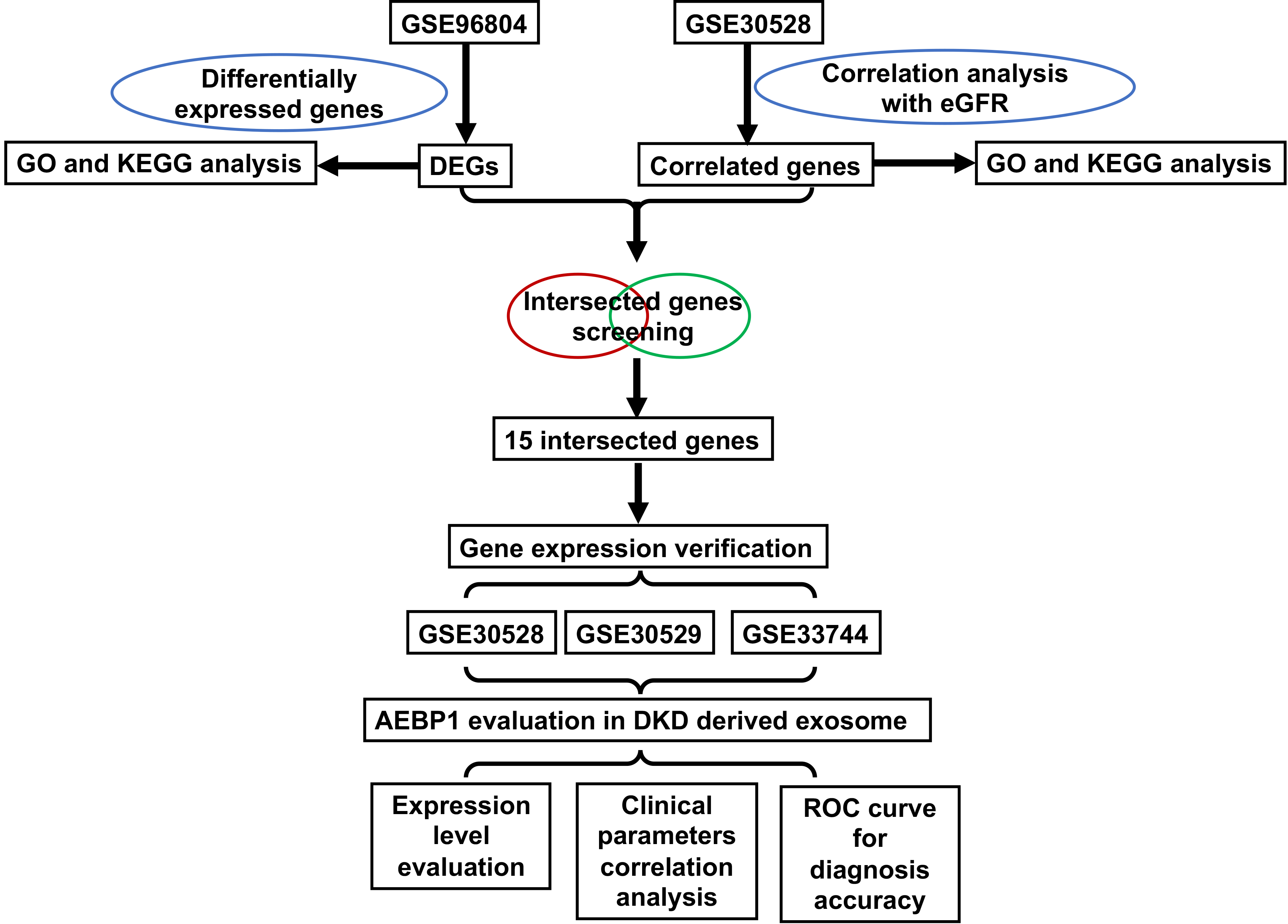

Supplement: Supplementary file 2 — Additional file 2: Figure S1. [file 12967_2021_3000_MOESM2_ESM.tif]

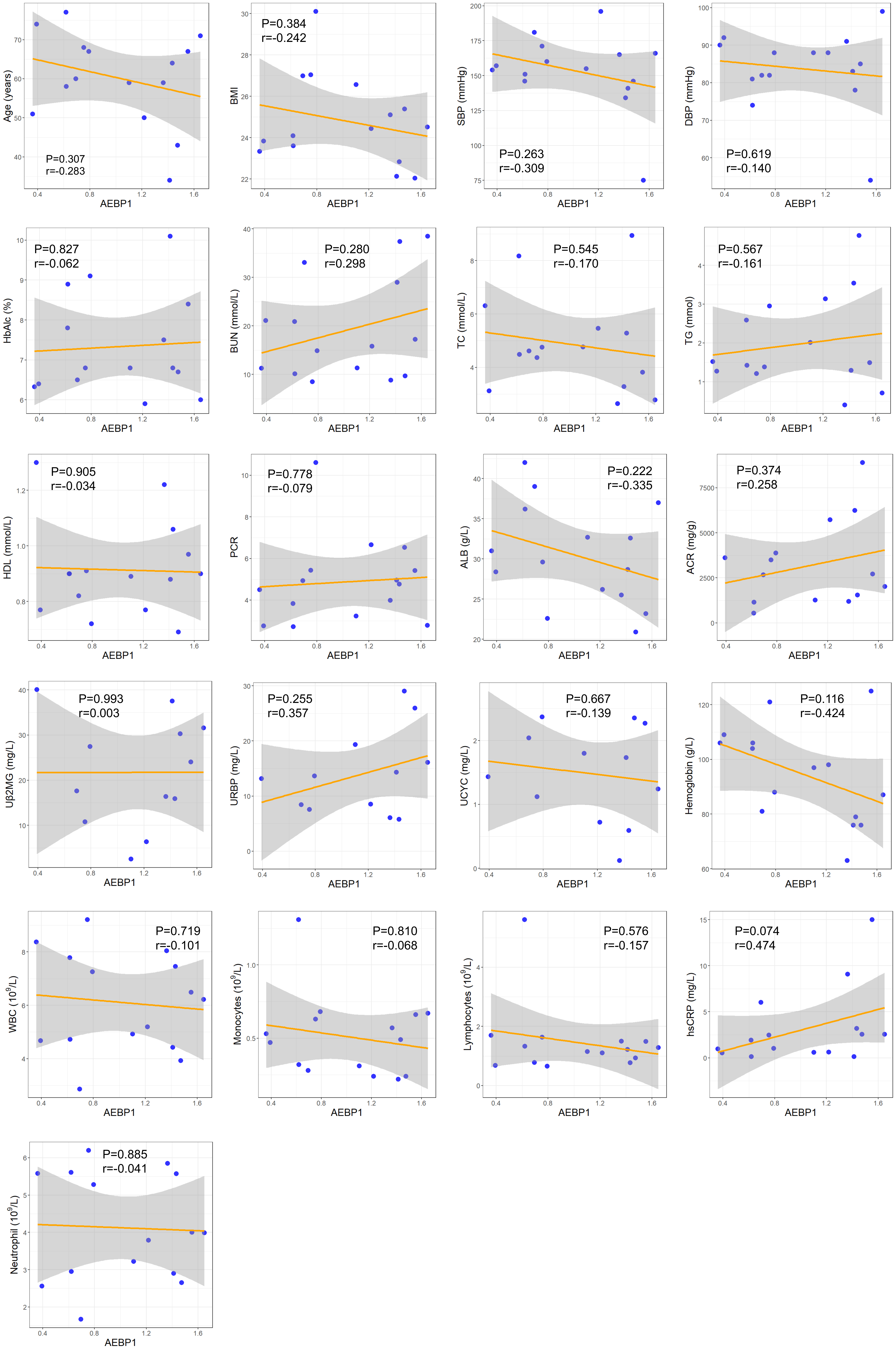

Supplement: Supplementary file 3 — Additional file 3: Figure S2. [file 12967_2021_3000_MOESM3_ESM.tif]

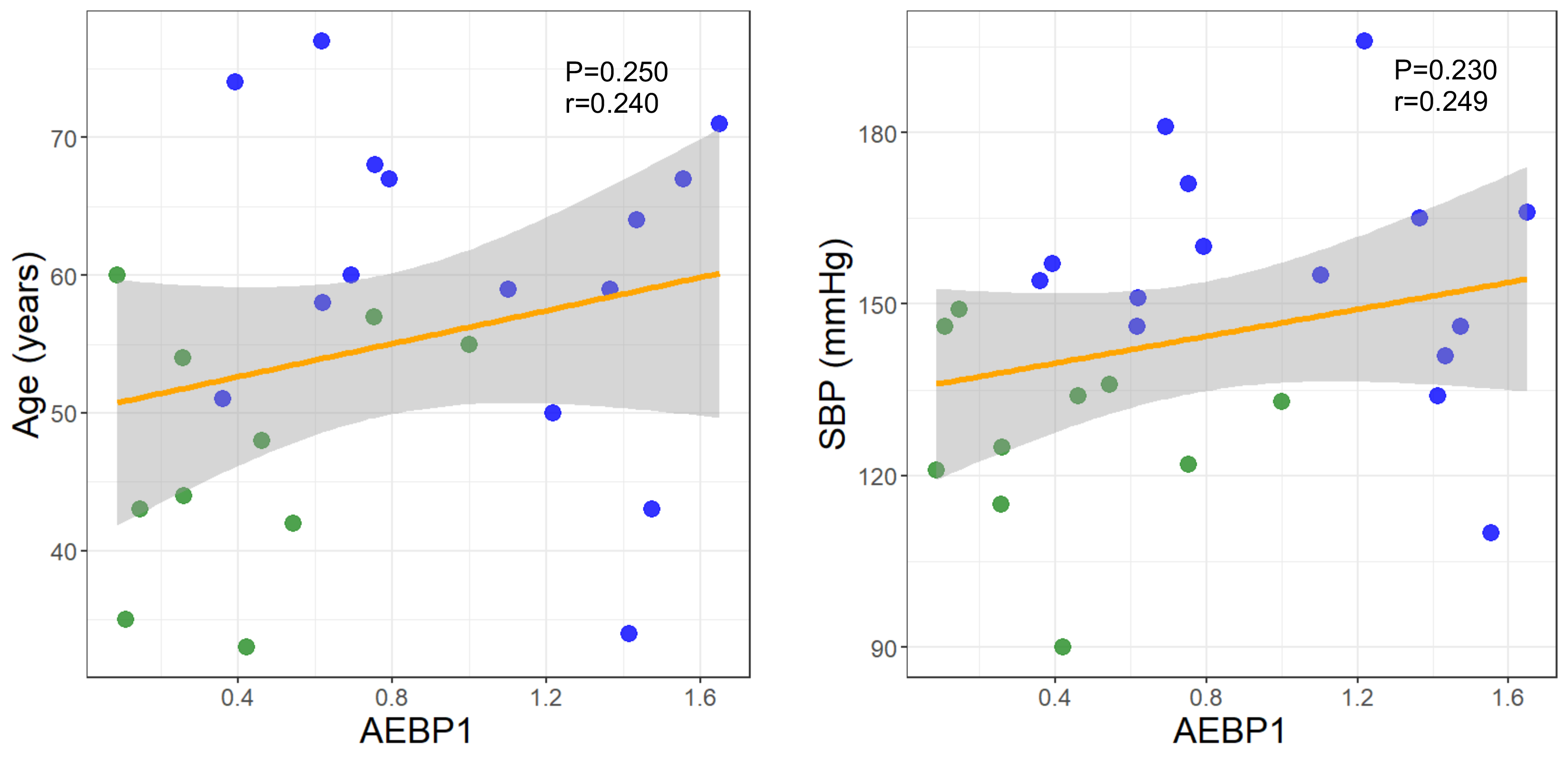

Supplement: Supplementary file 4 — Additional file 4: Figure S3. [file 12967_2021_3000_MOESM4_ESM.tif]
